# Chest computed tomography and multi-criteria decision analysis-guided interventions for stroke-associated pneumonia in acute ischemic stroke

**DOI:** 10.1590/1806-9282.20241871

**Published:** 2025-06-16

**Authors:** Weifeng Shu, Shumin Luo, Yuyu Zeng, Fengxin Yuan, Minjuan Ding, Zhimin He

**Affiliations:** 1 South China University of Technology, School of Medicine, The Sixth Affiliated Hospital, Department of Neurology – Foshan, China.

**Keywords:** Pneumonia, aspiration, Cerebral infarction, Decision support techniques, Tomography, X-ray computed, Care bundles

## Abstract

**OBJECTIVE:**

Stroke-associated pneumonia is a common complication of acute cerebral infarction, worsening prognosis and prolonging hospitalization. Early detection and timely intervention are critical to improving outcomes. The aim of this study was to evaluate the effectiveness of a multi-criteria decision analysis-based intervention in preventing stroke-associated pneumonia and examine the role of chest computed tomography in guiding early interventions.

**METHODS:**

A total of 77 patients with acute cerebral infarction were divided into a control group (n=38) receiving standard care and an intervention group (n=39) receiving an multi-criteria decision analysis-guided bundled intervention. Chest computed tomography identified early pulmonary infections, and interventions such as anti-infective and respiratory support were adjusted accordingly. Stroke-associated pneumonia incidence, hospital stay, and functional recovery were compared, and multivariate logistic regression analysis identified independent stroke-associated pneumonia risk factors.

**RESULTS:**

The intervention group showed a statistically significantly lower stroke-associated pneumonia incidence (12.8 vs. 31.6%, p=0.034), shorter hospital stays (14.1 vs. 16.8 days, p=0.027), and better improvements in Barthel Index for Activities of Daily Living (50.3 vs. 35.7, p=0.005) and Glasgow Coma Scale scores (14.1 vs. 12.7, p=0.041). Independent stroke-associated pneumonia risk factors included age (OR 1.03, 95%CI 1.01–1.06, p=0.029), Glasgow Coma Scale score (OR 0.83, 95%CI 0.73–0.94, p=0.014), and smoking history (OR 2.11, 95%CI 1.08–4.02, p=0.031). The intervention statistically significantly reduced stroke-associated pneumonia risk (OR 0.39, 95%CI 0.17–0.92, p=0.032).

**CONCLUSION:**

Multi-criteria decision analysis-guided interventions, supported by chest computed tomography for early detection, significantly reduce stroke-associated pneumonia incidence, improve recovery, and shorten hospital stays in acute cerebral infarction patients. Chest computed tomography is crucial for optimizing early interventions and treatment strategies.

## INTRODUCTION

Stroke-associated pneumonia (SAP) is a serious and prevalent complication in patients with acute ischemic stroke (AIS), significantly impacting clinical outcomes by increasing morbidity, prolonging hospitalization, and elevating mortality rates^1^. SAP typically occurs within the first few days after stroke onset due to factors such as impaired consciousness, dysphagia, and immobility, which predispose patients to aspiration and subsequent pulmonary infections^2^. SAP affects 10–30% of stroke patients, depending on the population and diagnostic criteria^3^. Its occurrence complicates stroke management, hinders functional recovery, and increases the likelihood of long-term disability, negatively affecting patients’ quality of life. Early identification and prevention of SAP are crucial for improving outcomes.

Traditional preventive measures for SAP, including early mobilization, respiratory hygiene, and prophylactic antibiotics, are often insufficient. Consequently, there is a growing need for comprehensive, evidence-based interventions tailored to individual risk profiles. Bundled interventions, which integrate multiple strategies targeting various risk factors, offer a promising approach. Such interventions, informed by individualized decision-making frameworks like multi-criteria decision analysis (MCDA), provide more holistic and effective solutions for high-risk patients. MCDA is a structured, systematic approach that integrates clinical variables and patient-specific characteristics, facilitating the formulation of personalized intervention strategies to optimize clinical outcomes^4^. For SAP prevention, MCDA can guide the design of tailored interventions that address each patient's unique risk profile, ensuring the application of the most relevant and timely measures.

In addition to implementing bundled interventions, early and accurate detection of pulmonary infections is essential. Chest computed tomography (CT) is a highly sensitive imaging modality capable of detecting subtle early changes, such as ground-glass opacities or consolidations, indicative of infection prior to clinical symptoms. Chest CT enables the timely adjustment of therapeutic strategies, including initiating or intensifying antimicrobial therapy and respiratory support, reducing SAP incidence and severity, and improving overall outcomes.

This study evaluated the effectiveness of an MCDA-guided bundled intervention for preventing SAP in AIS patients, incorporating chest CT for early detection of pulmonary infections and subsequent adjustments to therapeutic strategies. Multivariate logistic regression analysis was employed to identify independent SAP risk factors, providing further insights into individualized interventions. By combining the strengths of MCDA for personalized decision-making with the diagnostic sensitivity of chest CT, this research aimed to develop a more effective approach to SAP prevention. The findings may offer a valuable framework for improving AIS patient management, mitigating SAP risks, and enhancing clinical outcomes.

## METHODS

### Study design

This prospective, single-center, randomized controlled trial evaluated the effectiveness of an MCDA-guided bundled intervention for preventing SAP in AIS patients. The study compared outcomes between patients receiving standard care and those undergoing a personalized bundled intervention guided by early chest CT imaging. The study adhered to the Declaration of Helsinki and followed CONSORT guidelines.

### Study population

A total of 77 patients diagnosed with AIS were enrolled between January 2023 and January 2024. The inclusion criteria were: (1) confirmed AIS diagnosis based on clinical symptoms and neuroimaging (CT or magnetic resonance imaging); (2) age ≥18 years; and (3) ability to provide informed consent or consent by a legal representative. The exclusion criteria were: (1) pre-existing respiratory infection upon admission; (2) severe comorbidities such as advanced malignancy or end-stage renal disease; and (3) contraindications to chest CT or the proposed interventions. Participants were randomly assigned to intervention or control group using a computer-generated sequence.

### Interventions

The control group (n=38) received standard care, including routine monitoring, early mobilization, respiratory hygiene (e.g., incentive spirometry), and prophylactic antibiotics when indicated. Daily monitoring for pneumonia guided treatment adjustments per clinical guidelines.

**Figure 1:**
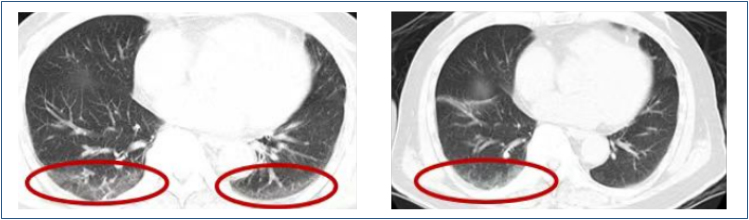
Early chest computed tomography findings in stroke-associated pneumonia detection. The chest computed tomography images show subclinical pulmonary infections. The circled areas highlight ground-glass opacities and early consolidations in the lower lobes of both lungs, which are indicative of the early signs of pneumonia.

The intervention group (n=39) received a bundled intervention guided by MCDA. Risk stratification considered factors such as age, Glasgow Coma Scale (GCS) score, and smoking history. Early chest CT imaging within 48 h of admission identified subclinical infections or SAP indicators like ground-glass opacities or consolidations (Figure 1). Targeted interventions included adjusted antimicrobial therapy and respiratory support (e.g., non-invasive ventilation), alongside enhanced airway management and supportive measures.

### Data collection

Baseline demographic and clinical data, including age, sex, body mass index (BMI), smoking history, and comorbidities (e.g., diabetes, hypertension, chronic lung disease, and atrial fibrillation), were collected. Neurological status was assessed with the GCS, and dysphagia was evaluated as an SAP risk factor. Functional status was measured using the Barthel Index for Activities of Daily Living (BADL). Imaging data were obtained via chest CT within 48 h to detect early pulmonary infection signs, with follow-up imaging as needed. Clinical outcomes such as SAP incidence, hospital stay length, and functional recovery were recorded. Adjustments in respiratory support or antimicrobial therapy based on imaging findings were documented.

### Outcome measures

The primary outcome was SAP incidence, diagnosed through clinical symptoms (fever, productive cough, and abnormal lung auscultation) and radiological evidence on follow-up chest CT or plain radiographs. Secondary outcomes included hospital stay length (admission to discharge) and functional recovery, measured by BADL scores. Neurological recovery was assessed through GCS score changes, tracking consciousness and motor function improvements during hospitalization. Outcomes were compared between the groups to assess the intervention's effectiveness.

### Statistical analysis

Descriptive statistics summarized baseline characteristics, using means and standard deviations for continuous variables and frequencies for categorical variables. SAP incidence was compared between groups using the chi-square test or Fisher's exact test. Continuous variables (e.g., hospital stay length, BADL, and GCS scores) were analyzed using independent samples t-test or Mann-Whitney U test. Multivariate logistic regression analysis identified independent SAP predictors, with variables selected based on clinical relevance and statistical significance in univariate analysis. Odds ratios (ORs) with 95% confidence intervals (CIs) quantified associations between predictors and SAP. A two-sided p-value <0.05 was considered statistically significant. Analyses were conducted using SPSS v. 26.0 software (IBM, Armonk, NY, USA).

## RESULTS

### Baseline characteristics

A total of 77 patients were included in the study, with 38 in the control group and 39 in the intervention group. Baseline characteristics such as age, sex, GCS score, smoking history, and pre-existing comorbidities (e.g., diabetes and chronic obstructive pulmonary disease) were recorded. Additionally, BMI, a history of hypertension, atrial fibrillation, and dysphagia upon admission were assessed. There were no statistically significant differences between the two groups regarding these baseline characteristics, ensuring that the groups were comparable at the start of the study. The detailed baseline characteristics are presented in Table 1.

**Table 1:** Baseline characteristics of the study population.

Characteristics	Control group (n=38)	Intervention group (n=39)	P
Age (years), mean±SD	68.5±9.4	69.1±8.7	0.721
Male, n (%)	21 (55.3)	23 (59.0)	0.781
GCS score	13.1±1.2	13.3±1.1	0.659
Smoking history, n (%)	12 (31.6)	13 (33.3)	0.864
Diabetes, n (%)	10 (26.3)	11 (28.2)	0.820
Chronic lung disease, n (%)	8 (21.1)	9 (23.1)	0.803
BMI (kg/m²), mean±SD	25.4±3.2	25.7±3.1	0.684
Hypertension, n (%)	24 (63.2)	25 (64.1)	0.917
Atrial fibrillation, n (%)	9 (23.7)	10 (25.6)	0.841
Dysphagia on admission, n (%)	13 (34.2)	14 (35.9)	0.882

GCS: Glasgow Coma Scale; BMI: Body mass index; SD: standard deviation.

### Primary outcome: stroke-associated pneumonia incidence

The incidence of SAP was significantly lower in the intervention group compared to the control group. In the intervention group, 5 patients (12.8%) developed SAP, whereas 12 patients (31.6%) in the control group were diagnosed with SAP. The difference between the two groups was statistically significant (p=0.034), indicating that the MCDA-based bundled intervention, guided by early detection via chest CT, was effective in reducing the risk of SAP.

### Secondary outcomes

The length of hospital stay was significantly shorter in the intervention group compared to the control group. Patients in the intervention group had an average hospital stay of 14.1±3.4 days, while those in the control group had an average stay of 16.8±4.2 days (p=0.027). This demonstrates that the intervention group benefited from a more rapid recovery and discharge.

Functional recovery, assessed using the BADL, showed a significant improvement in the intervention group compared to the control group. The intervention group had a mean BADL score of 50.3±7.8 at discharge, whereas the control group had a mean score of 35.7±9.2 (p=0.005). This suggests that patients in the intervention group regained greater independence in daily activities.

Similarly, neurological recovery, evaluated using the GCS score, showed significant improvement in the intervention group. The mean GCS score at discharge was 14.1±0.9 in the intervention group compared to 12.7±1.2 in the control group (p=0.041), indicating better overall neurological function.

### Univariate and multivariate logistic regression analyses of risk factors for stroke-associated pneumonia

Univariate analysis was performed to identify potential factors associated with SAP. The analysis revealed that age, GCS score, smoking history, dysphagia on admission, atrial fibrillation, chronic lung disease, and BMI were statistically significantly associated with SAP (p<0.05) (Table 2). These variables were subsequently included in the multivariate logistic regression model to determine the independent predictors of SAP. The multivariate analysis identified three independent risk factors for SAP: age (OR 1.03, 95%CI 1.01–1.06, p=0.029), GCS score (OR 0.83, 95%CI 0.73–0.94, p=0.014), and smoking history (OR 2.11, 95%CI 1.08–4.02, p=0.031). Additionally, the intervention strategy was found to statistically significantly reduce the risk of SAP (OR 0.39, 95%CI 0.17–0.92, p=0.032), further supporting the effectiveness of the MCDA-guided bundled intervention, combined with early detection via chest CT, in lowering the incidence of SAP (Table 2).

**Table 2:** Univariate and multivariate logistic regression analyses of risk factors for stroke-associated pneumonia.

Variables	Univariate OR (95%CI)	p	Multivariate OR (95%CI)	p
Age (per year)	1.04 (1.01–1.07)	0.020*	1.03 (1.01–1.06)	0.029*
GCS score	0.81 (0.71–0.92)	<0.001*	0.83 (0.73–0.94)	0.014*
Smoking history	2.36 (1.28–4.38)	0.023*	2.11 (1.08–4.02)	0.031*
Dysphagia on admission	2.46 (1.17–5.36)	0.038*	2.22 (0.98–5.02)	0.069
BMI (per kg/m²)	0.91 (0.85–0.98)	0.049*	0.93 (0.86–1.02)	0.087
Diabetes	1.59 (0.77–3.25)	0.191	–	–
Hypertension	1.43 (0.73–2.95)	0.222	–	–
Atrial fibrillation	2.73 (1.20–6.15)	0.025*	2.35 (1.02–5.39)	0.061
Chronic lung disease	3.05 (1.26–7.31)	0.016*	2.64 (1.04–6.73)	0.054
Intervention group	0.37 (0.16–0.80)	0.014*	0.39 (0.17–0.92)	0.032*

OR: odds ratio; CI: confidence interval; GCS: Glasgow Coma Scale; BMI: body mass index.

* p<0.05 indicates statistical significance.

## DISCUSSION

This study demonstrates that an MCDA-guided bundled intervention, combined with early chest CT detection, significantly reduced the incidence of SAP in patients with AIS. The findings validate the hypothesis that early identification of pulmonary infections via chest CT, coupled with personalized preventive measures tailored to patient-specific risk factors, can enhance clinical outcomes by reducing SAP occurrence. Beyond reducing SAP incidence, the intervention also improved functional and neurological recovery and shortened hospital stays.

Multivariate logistic regression analysis identified three independent risk factors for SAP: age, GCS score, and smoking history. Older age, a well-known risk factor for pneumonia, is linked to immunosenescence and increased vulnerability to respiratory infections. This study confirmed that advancing age significantly heightened SAP risk, aligning with the existing literature. Similarly, lower GCS scores, indicative of impaired consciousness, were strongly associated with SAP, as reduced consciousness increases aspiration risk—a major contributor to pneumonia in stroke patients. Smoking history further emerged as a significant risk factor, emphasizing the negative effects of chronic smoking on pulmonary function and immune response^5^.

Univariate analysis also revealed that dysphagia, atrial fibrillation, chronic lung disease, and lower BMI were associated with SAP. However, these variables did not achieve statistical significance in the multivariate model, likely due to confounding effects from stronger predictors, such as GCS score and smoking history. Dysphagia, while not independently significant in the multivariate analysis, remains clinically critical as a major contributor to aspiration pneumonia^6^.

The intervention strategy, incorporating early chest CT screening and MCDA-guided individualized measures, effectively reduced SAP risk. Patients in the intervention group experienced significantly lower SAP incidence compared to those receiving standard care. Chest CT enabled early detection of subclinical pulmonary infections, allowing for timely adjustments in antimicrobial therapy and respiratory support, which prevented progression to full-blown pneumonia^7^. These results underscore the utility of chest CT as a sensitive diagnostic tool for early pulmonary complications in stroke patients.

The implementation of an MCDA-guided intervention highlights the value of personalized preventive strategies. MCDA integrates multiple clinical variables into the decision-making process, enabling the development of tailored interventions that address patient-specific risk profiles^8^. This personalized approach contrasts with conventional, generalized preventive strategies and is particularly advantageous for high-risk populations such as AIS patients, in whom pneumonia risk factors vary widely.

The findings also emphasize the importance of addressing modifiable risk factors, such as smoking, in SAP management. Smoking cessation programs should be integrated into post-stroke care, especially for patients with chronic lung disease or elevated risk of pulmonary infections. Additionally, improving GCS scores through early rehabilitation and neurological support can reduce aspiration risk and further lower SAP incidence.

Despite the encouraging findings, several limitations must be acknowledged. This study was conducted at a single center with a relatively small sample size, which may limit the generalizability of the results. Larger, multi-center studies are necessary to confirm these findings and evaluate the intervention's applicability across diverse clinical settings. Furthermore, while chest CT was the primary imaging modality for early pneumonia detection, its routine use in all stroke patients may not be feasible due to cost and resource constraints, as well as the need for frequent imaging in certain cases.

Another limitation is the relatively short follow-up period. This study focused on the acute phase of stroke care, and long-term follow-up would provide valuable insights into the durability of the intervention's effects on functional recovery and SAP incidence beyond initial hospitalization. Future research should explore the long-term impact of early SAP prevention on functional outcomes and quality of life in stroke survivors. Preventing pneumonia during early stroke recovery may have enduring benefits, reducing disability burden and improving rehabilitation outcomes.

In conclusion, this study demonstrates that an MCDA-guided bundled intervention, supported by early chest CT detection, is highly effective in reducing SAP incidence in AIS patients. The intervention also improved functional and neurological recovery and shortened hospital stays. Independent SAP risk factors—age, GCS score, and smoking history—highlight the importance of individualized preventive strategies. These findings provide a strong rationale for incorporating advanced decision-making tools and imaging techniques into the routine management of high-risk stroke patients to improve clinical outcomes.
